# Prevalence of Elective and Ruptured Abdominal Aortic Aneurysm Repairs by Age and Sex From 2003 to 2016 in Ontario, Canada

**DOI:** 10.1001/jamanetworkopen.2018.5418

**Published:** 2018-11-30

**Authors:** Konrad Salata, Mohamad A. Hussain, Charles de Mestral, Elisa Greco, Muhammad Mamdani, Thomas L. Forbes, Deepak L. Bhatt, Subodh Verma, Mohammed Al-Omran

**Affiliations:** 1 Department of Surgery, Division of Vascular Surgery, University of Toronto, Toronto, Ontario, Canada; 2 Division of Vascular Surgery, Li Ka Shing Knowledge Institute of St Michael’s Hospital, Toronto, Ontario, Canada; 3 Li Ka Shing Centre for Healthcare Analytics Research and Training, Li Ka Shing Knowledge Institute, St Michael’s Hospital Toronto, Ontario, Canada; 4 Leslie Dan Faculty of Pharmacy, University of Toronto, Toronto, Ontario, Canada; 5 Department of Medicine, Faculty of Medicine, University of Toronto, Toronto, Ontario, Canada; 6 Institute of Health Policy, Management and Evaluation, Dalla Lana Faculty of Public Health, University of Toronto, Toronto, Ontario, Canada; 7 Division of Vascular Surgery, Peter Munk Cardiac Centre, University Health Network, Toronto, Ontario, Canada; 8 Brigham and Women's Hospital Heart and Vascular Center, Boston, Massachusetts; 9 Harvard Medical School, Boston, Massachusetts; 10 Department of Surgery, Division of Cardiac Surgery, University of Toronto, Toronto, Ontario, Canada; 11 Division of Cardiac Surgery, Li Ka Shing Knowledge Institute of St Michael’s Hospital, Toronto, Ontario, Canada; 12 Department of Surgery, King Saud University, Riyadh, Kingdom of Saudi Arabia

## Abstract

**Importance:**

Age and sex are important considerations in assessing and individualizing therapy for abdominal aortic aneurysm (AAA) repair.

**Objective:**

To determine the prevalence of open and endovascular elective AAA (EAAA) and ruptured AAA (RAAA) repair by age and sex.

**Design, Setting, and Participants:**

In this retrospective, population-based, cross-sectional, time-series analysis in Ontario, Canada, from April 1, 2003, to March 31, 2016, all patients undergoing AAA repair who were older than 39 years were included.

**Exposures:**

Elective AAA and RAAA repair with open surgical repair (OSR) or endovascular aortic repair (EVAR).

**Main Outcomes and Measures:**

Age- and sex-standardized rates of EAAA and RAAA repair with OSR and EVAR.

**Results:**

From 2003 to 2016, 19 489 EAAA repairs (12 232 [63%] OSR and 7257 [37%] EVAR) and 2732 RAAA repairs (2466 [90%] OSR and 266 [10%] EVAR) were identified. The mean (SD) age was 72.7 (8.1) years in the EAAA subgroup and 73.5 (8.9) years in the RAAA subgroup; 15 813 patients (81%) in the EAAA subgroup and 2178 (80%) in the RAAA subgroup were men. The rates of EAAA by age quintile and sex decreased over the study period except among patients older than 79 years (1.3 per 100 000 population in 2003 to 2.2 per 100 000 population in 2016; 70% increase; *P* < .001). The rates of elective OSR decreased across all age and sex subgroups (range, 38%-74% decrease; *P* ≤ .009 for all subgroups) except among patients older than 79 years (1.3 per 100 000 population at baseline to 0.56 per 100 000 population in the second quarter of 2016; 53% decrease; *P* = .05). The rates of elective EVAR significantly increased across all age and sex subgroups (range, 566%-1585% increase; *P* ≤ .04 for all subgroups). Elective EVAR became the dominant treatment approach for aneurysms in men around 2010, whereas it maintained parity among women in 2016. The RAAA repair rate decreased over the study period in all subgroups (range, 32%-91% decrease; *P* ≤ .001 for all subgroups), but the decrease was not significant among women (80% decrease; *P* = .08). Similarly, the rates of ruptured OSR decreased among all subgroups (range, 47%-91% decrease; *P* < .001), but the decrease was not significant among women (87% decrease; *P* = .54). Ruptured EVAR showed significant uptake in all subgroups.

**Conclusions and Relevance:**

Among patients with AAA in Ontario, Canada, use of EVAR appeared to increase from 2003 to 2016, whereas OSR use appeared to decrease. These findings were most pronounced among elective procedures for men and older patients. The delayed increase in the use of EVAR among women may reflect continued anatomical constraints for women seeking elective repair.

## Key Points

**Question:**

What was the prevalence of elective and ruptured abdominal aortic aneurysm repairs by age and sex from 2003 to 2016 in Ontario, Canada?

**Findings:**

This population-based, cross-sectional study of 19 489 elective and 2732 ruptured abdominal aortic aneurysm repairs revealed an increase in the rate of elective abdominal aortic aneurysm repair among patients older than 79 years. Endovascular repair was the preferred treatment approach for elective repair among men, although there was greater uptake for ruptured repair among women.

**Meaning:**

The findings suggest that endovascular repair may be the preferred method for elective and ruptured aneurysm repair, particularly among older individuals, for elective repairs in men, and for ruptured repairs in women.

## Teaser

This cross-sectional, time-series analysis examines the prevalence of open and endovascular elective and ruptured abdominal aortic aneurysm repairs by age and sex from 2003 to 2016 in Ontario, Canada.

## Introduction

Age and sex are well-established risk factors for many cardiovascular diseases, including abdominal aortic aneurysms (AAAs). The increasing prevalence with age and the 4:1 male to female ratio of AAA prevalence are both well established in the literature.^1,2,3,4^ Furthermore, these demographics are related to anatomic and physiological considerations that influence the approach to AAA treatment.

The approach to AAA treatment requires a careful consideration of comorbidities and correspondent perioperative mortality and morbidity risk, weighed against the natural history of the untreated aneurysm. The correlation among age, cardiopulmonary comorbidity, and perioperative mortality is well known, such that age is a common covariate included in perioperative risk prediction models.^5^ Before the introduction of endovascular aortic repair (EVAR), elderly patients were frequently turned down even for elective AAA (EAAA) repair owing to the prohibitive surgical risk associated with conventional open surgical repair (OSR).^6^ The lower perioperative mortality associated with EVAR changed the approach to treatment of EAAA and ruptured AAA (RAAA), providing patients with previously inoperable AAA with a treatment option.^6,7^

Sex introduces additional technical considerations to the treatment of vascular disease. The application of EVAR to treatment of AAA in women was partially limited by a size mismatch between the size of the endograft delivery system and the smaller size of femoral and iliac arteries in women.^8^ However, refinements in endograft fixation and stent organization have led to reductions in delivery system size and expansion of available endograft diameters. Current delivery systems have outer diameters as small as 16 French. Thus, over time, the application of EVAR for AAA in the female anatomic structure should be less limited by available technology.

Understanding variable trends in the application of EVAR for AAA treatment by age group and sex may help to clarify and address limitations in EVAR technology. This retrospective, population-based, time-series analysis study sought to determine whether age and sex differences in the application of EVAR for the treatment of AAA exist by analyzing the age- and sex-specific rates of elective OSR, elective EVAR, ruptured OSR, and ruptured EVAR over 13 years (2003-2016).

## Methods

### Study Design and Setting

This cross-sectional study was approved by the institutional review board at Sunnybrook Health Sciences Centre, Toronto, Ontario, Canada. Informed consent of study participants was waived because this study used secondary health information that was deidentified. This retrospective, population-based, cross-sectional, time-series analysis of OSR and EVAR of EAAA and RAAA in Ontario, Canada, was conducted in accordance with the Strengthening the Reporting of Observational Studies in Epidemiology (STROBE) reporting guideline for cross-sectional studies.^9^ Ontario is Canada’s most populous province, with more than 13 million residents. All Ontarians with a valid health card have access to single-payer, publicly funded health care.

### Data Sources

All publicly insured ambulatory, emergency, and inpatient health care system interactions requiring the use of an Ontario health card are recorded by the Ministry of Health and Long-Term Care of Ontario for the purposes of system assessment and planning. These data are stored and managed by the Institute for Clinical Evaluative Sciences (ICES), a prescribed entity governed under the Personal Health Information Protection Act. The ICES data are derived from multiple primary data sources provided by the federal and provincial governments as well as various research organizations, registries, and initiatives. These data are anonymized and linked using an ICES key number. The specific data sets used for this study include the Canadian Institute for Health Information Discharge Abstract Database and Same Day Surgery Database, the National Ambulatory Care Reporting System database, and the Ontario Health Insurance Plan database.^10^

### Patient Cohort

The study cohort consisted of all Ontarians older than 39 years who underwent EAAA and RAAA repair in Ontario, Canada, from April 1, 2003, to March 31, 2016. Patients receiving elective OSR, ruptured OSR, elective EVAR, and ruptured EVAR were identified using a combination of the *International Statistical Classification of Diseases and Related Health Problems, Tenth Revision, Canada* (*ICD-10-CA*), *Canadian Classification of Health Intervention* (*CCI*), and Ontario Health Insurance Plan codes according to a previously validated algorithm.^10^ Sex and age were collected directly from the described databases.

### Statistical Analysis

The study period was divided into 52 quarterly intervals from April 1, 2003, to March 31, 2016. The number of overall and approach-specific EAAA and RAAA repairs conducted during each quarterly interval were counted. Observed quarterly age- and sex-specific repair rates per 100 000 population were calculated using the quarterly, age quintile–specific Ontario population or the quarterly, sex-specific Ontario population older than 39 years as the denominator. The denominators for rate calculations were derived from the 2015 Canadian census data and associated projections. Age quintiles were defined as 40 to 64 years (quintile 1), 65 to 69 years (quintile 2), 70 to 74 years (quintile 3), 75 to 79 years (quintile 4), and older than 79 years (quintile 5).

Where possible, the percent change in repair rate was calculated for each group relative to the repair rate in the second quarter of 2003 (baseline). To examine the presence of statistically significant trends within each group, autoregressive integrated moving average (ARIMA) models were fit with linear or quadratic trend regressors as dictated by visual inspection of graphical data. ARIMA models are specific applications of linear regression models for time-series data with autocorrelated errors.^11,12^ Model appropriateness was assessed using autocorrelation, partial and inverse autocorrelation plots, and the Ljung-Box *q* statistic. Model fit was evaluated using the Akaike information criterion and the adjusted *R*^2^ values. All statistical analyses were conducted in SAS, version 9.4 (SAS Institute), with a 2-sided *P* value of less than .05 for statistical significance.

## Results

### Cohort Characteristics

Among patients from Ontario administrative data from 2003 to 2016, a total of 19 489 EAAA repairs (12 232 elective OSRs [63%] and 7257 elective EVARs [37%]) and 2732 RAAA repairs (2466 ruptured OSRs [90%] and 266 ruptured EVARs [10%]) were identified (Table). The mean (SD) age was 72.7 years (8.1) in the EAAA subgroup and 73.5 (8.9) in the RAAA subgroup. Most patients were men (15 813 [81%] in the EAAA subgroup and 2178 [80%] in the RAAA subgroup).

**Table.** Characteristics of Overall and Approach-Specific Elective and Ruptured Abdominal Aortic Aneurysm Repair Cohorts in Ontario, Canada, 2003-2016

Variable	Abdominal Aortic Aneurysm Repair, No. (%)			
	Elective (n = 19 489)		Ruptured (n = 2732)	
	Open Surgical Repair (n = 12 232)	Endovascular Aortic Repair (n = 7257)	Open Surgical Repair (n = 2466)	Endovascular Aortic Repair (n = 266)
Age, mean (SD), y	71.3 (7.8)	75.2 (7.9)	73.4 (8.8)	74.6 (9.8)
Age quintile, y				
40-64	2397 (20)	732 (10)	426 (17)	40 (15)
65-69	2425 (20)	997 (14)	371 (15)	38 (14)
70-74	2909 (24)	1430 (20)	520 (21)	48 (18)
75-79	2690 (22)	1772 (24)	483 (20)	55 (21)
>79	1811 (15)	2326 (32)	666 (27)	85 (32)
Sex				
Men	9662 (79)	6151 (85)	1963 (80)	215 (81)
Women	2570 (21)	1106 (15)	503 (20)	51 (19)

### Age- and Sex-Specific Rates of EAAA Repair

**Figure 1.**
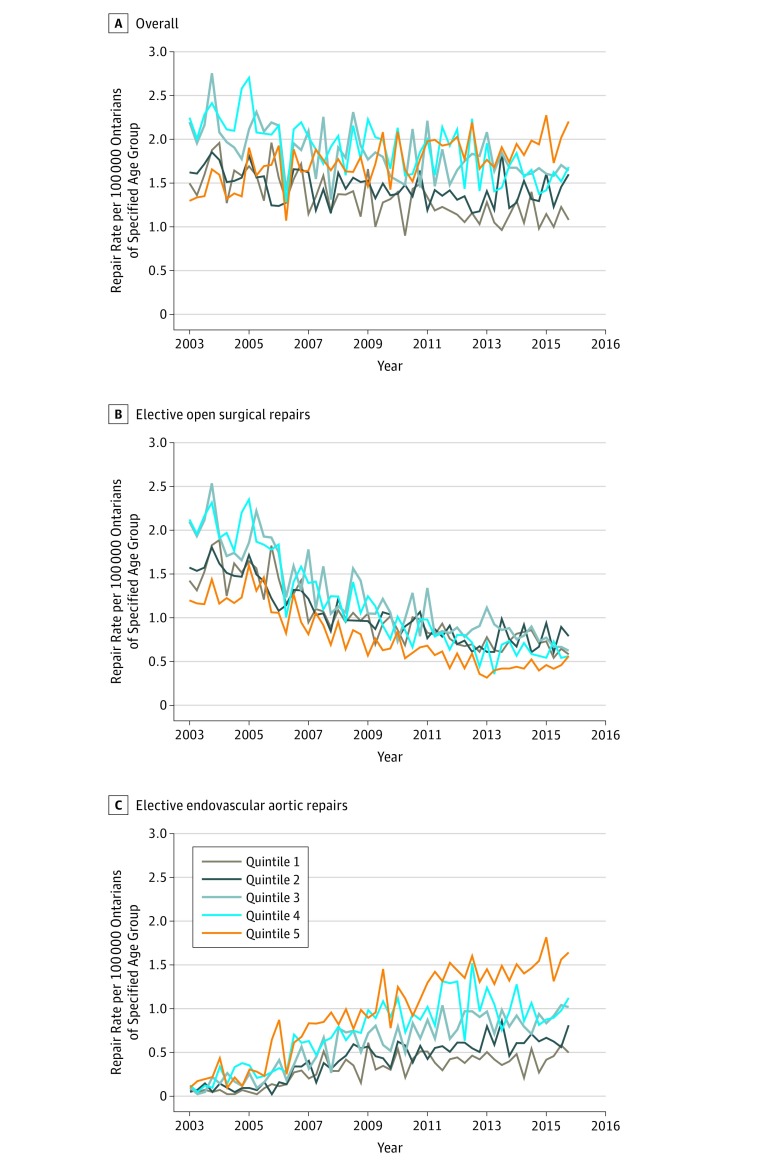
Overall and Approach-Specific Elective Abdominal Aortic Aneurysm Repair Rates by Age Quintile in Ontario, Canada, 2003-2016 Age quintile definitions: quintile 1 included patients aged 40 to 64 years; quintile 2, patients aged 65 to 69 years; quintile 3, patients aged 70 to 74 years; quintile 4, patients aged 75 to 79 years; and quintile 5, patients older than 79 years. *P* values represent statistical significance of coefficient for trend regressor from the autoregressive integrated moving average model and indicate the presence of a statistically significant trend. A, For quintiles 1 through 5, *P* < .001. B, Quintile 1, *P* = .009; quintiles 2, 3, and 4, *P* < .001; and quintile 5, *P* = .05. C, Quintile 1, *P* = .04; quintile 2, *P* = .04; quintile 3, *P* = .01; and quintiles 4 and 5, *P* < .001.

The overall age quintile–specific rates of EAAA repair decreased over the study period and demonstrated negative trends (*P* < .001 for all quintiles) (Figure 1A). Quintile 1 demonstrated the greatest decrease in EAAA repair rate per 100 000 population (1.49 at baseline to 1.08 in the second quarter of 2016 [28% decrease]; *P* < .001), whereas quintile 2 had the smallest decrease (1.62 at baseline to 1.60 in the second quarter of 2016 [1% decrease]; *P* < .001). The rate of EAAA repair per 100 000 population in quintile 5 increased over the study period (1.3 at baseline to 2.2 in the second quarter of 2016 [70% increase]; *P* < .001).

All age quintiles demonstrated significant negative trends for elective OSRs (range, 38%-74% decrease; *P* ≤ .009 for all subgroups) except for quintile 5 (1.3 per 100 000 population at baseline to 0.56 per 100 000 population in the second quarter of 2016; 53% decrease; *P* = .05), whereas all elective EVAR quintiles demonstrated significant positive trends (*P* ≤ .04) (Figure 1B and C). Over the study period, the greatest decrease in the rate of elective OSR per 100 000 population was seen in quintile 4 (1.42 at baseline to 0.56 in the second quarter of 2016 [74% decrease]; *P* < .001), whereas quintile 2 had the smallest decrease (1.57 at baseline to 0.78 in the second quarter of 2016 [50% decrease]; *P* < .001). Among elective EVARs, quintile 5 rates per 100 000 population demonstrated the greatest increase (0.10 at baseline to 1.64 in the second quarter of 2016 [1545% increase]; *P* < .001), whereas quintile 1 demonstrated the smallest increase (0.07 at baseline to 0.49 in the second quarter of 2016 [566% increase]; *P* = .04).

**Figure 2.**
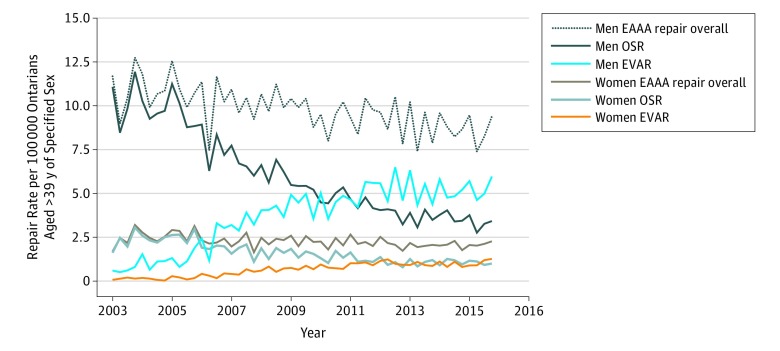
Overall and Approach-Specific Elective Abdominal Aortic Aneurysm (EAAA) Repair Rates by Sex in Ontario, Canada, 2003-2016 *P* values represent statistical significance of coefficient for trend regressor from the autoregressive integrated moving average model and indicate the presence of a statistically significant trend. For men undergoing EAAA overall, by elective open surgical repair (OSR), and by elective endovascular aortic repair (EVAR), *P* < .001. For women undergoing EAAA repair overall and by elective OSR, *P* < .001; by EVAR, *P* = .006.

Rates of overall EAAA repair among men and women demonstrated significant negative (*P* < .001) and positive (*P* < .001) trends, respectively (Figure 2). The rate of overall EAAA repair per 100 000 population among men decreased from 11.68 at baseline to 9.40 in the second quarter of 2016 (20% decrease; *P* < .001), whereas this rate increased from 1.69 at baseline to 2.27 in the second quarter of 2016 for women (34% increase; *P* < .001). The elective OSR rate per 100 000 population for men decreased over the study period (11.08 at baseline to 3.43 in the second quarter of 2016 [69% decrease]; *P* < .001 for trend), as did the elective OSR rate for women (1.62 at baseline to 1.00 in the second quarter of 2016 [38% decrease]; *P* < .001). In contrast, the elective EVAR rates per 100 000 population for both men and women increased (men, 0.60 at baseline to 5.96 in the second quarter of 2016 [889% increase]; *P* < .001 for trend; women, 0.08 at baseline to 1.27 in the second quarter of 2016 [1585% increase]; *P* = .006 for trend). For men, EVAR became the predominant approach to EAAA repair around 2010, whereas the proportion of EAAAs repaired by EVAR in women reached and remained approximately 50% around 2012.

### Age- and Sex-Specific Rates of RAAA Repair

**Figure 3.**
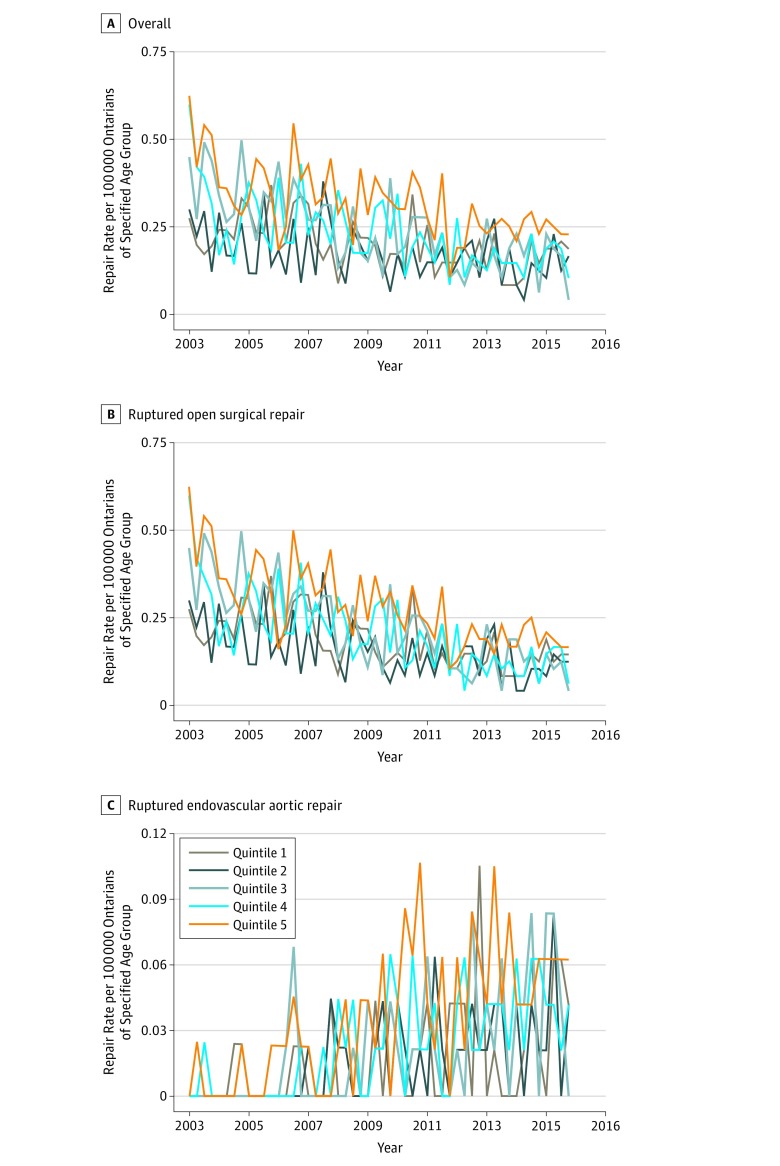
Overall and Approach-Specific Ruptured Abdominal Aortic Aneurysm Repair Rates by Age Quintile in Ontario, Canada, 2003-2016 Age quintile definitions: quintile 1 included patients aged 40 to 64 years; quintile 2, patients aged 65 to 69 years; quintile 3, patients aged 70 to 74 years; quintile 4, patients aged 75 to 79 years; and quintile 5, patients older than 79 years. *P* values represent statistical significance of coefficient for trend regressor from the autoregressive integrated moving average model and indicate the presence of a statistically significant trend. A, For quintile 1, *P* = .001, and for quintiles 2 through 5, *P* < .001. B, For quintiles 1 through 5, *P* < .001. C, For quintiles 1 through 5, *P* < .001.

The overall age quintile–specific rates of RAAA repair decreased and demonstrated significant negative trends over the study period (*P* ≤ .001 for all age quintiles) (Figure 3A). The greatest decrease in overall RAAA repair rate per 100 000 population was seen in age quintile 3, in which the rate of RAAA repair decreased from 0.45 at baseline to 0.04 in the second quarter of 2016 (91% decrease; *P* < .001). Quintile 1 had the smallest decrease in overall RAAA repair rate per 100 000 population (0.27 at baseline to 0.19 in the second quarter of 2016 [32% decrease]; *P* < .001).

Regarding the approach-specific rates, the rates of ruptured OSR decreased, whereas ruptured EVAR rates increased across all age quintiles (Figure 3B and C). All ruptured OSR and ruptured EVAR rates demonstrated significant trends (*P* < .001 for all quintiles). Mirroring the overall RAAA repair rate changes, the ruptured OSR repair rate per 100 000 population for quintile 3 demonstrated the greatest ruptured OSR decrease (0.45 at baseline to 0.04 in the second quarter of 2016 [91% decrease]), and quintile 1 demonstrated the smallest decrease (0.27 at baseline to 0.15 in the second quarter of 2016 [47% decrease]). Among ruptured EVARs, quintile 5 demonstrated the greatest rate increase per 100 000 population (0/100 000 at baseline to 0.06 in the second quarter of 2016), whereas quintiles 1 through 4 demonstrated smaller absolute increases (0 at baseline to 0.04 in the second quarter of 2016).

**Figure 4.**
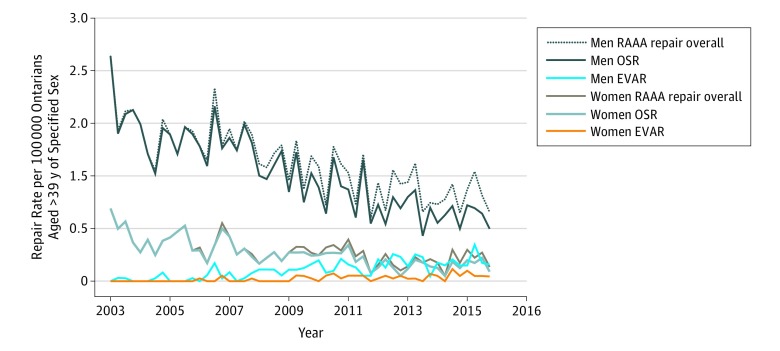
Overall and Approach-Specific Ruptured Abdominal Aortic Aneurysm (RAAA) Repair Rates by Sex in Ontario, Canada, 2003-2016 *P* values represent statistical significance of coefficient for trend regressor from the autoregressive integrated moving average model and indicate the presence of statistically significant trend. For men undergoing RAAA repair overall, by open surgical repair (OSR), and by endovascular aortic repair (EVAR), *P* < .001. For women undergoing RAAA repair overall, *P* = .08; by OSR, *P* = .54; and by EVAR, *P* < .001.

The rates of overall RAAA repair per 100 000 population among men and women decreased (men, 2.56 at baseline to 0.79 in the second quarter of 2016 [69% decrease]; women, 0.83 at baseline to 0.16 in the second quarter of 2016 [80% decrease]). The RAAA repair rates for men demonstrated a significant negative trend (*P* < .001), whereas rates for women demonstrated no significant trends (*P* = .08) (Figure 4). Likewise, the ruptured OSR rates per 100 000 population for both men and women decreased (men, 2.56 at baseline to 0.65 in the second quarter of 2016 [75% decrease]; women, 0.83 at baseline to 0.12 in the second quarter of 2016 [87% decrease]), but only the rates for men showed a significant negative trend (*P* < .001 for men; *P* = .54 for women). The ruptured EVAR rates per 100 000 population for men and women increased over the study period (men, 0 at baseline to 0.20 in the second quarter of 2016; women, 0 at baseline to 0.06 in the second quarter of 2016). Both groups showed significant positive sex-specific ruptured EVAR trends (*P* < .001 for both).

## Discussion

This population-based time-series analysis found significant age- and sex-specific trends in overall and approach-specific EAAA and RAAA repair rates from 2003 to 2016. Of note, the study showed that EAAA repair rates increased in patients older than 79 years in association with an increase in the use of EVAR within this age group. Furthermore, EVAR became the predominant approach over OSR for EAAA repair in men around 2010, whereas its rate of use was similar to that of OSR around 2012 in women. Finally, this study showed that EVAR uptake increased among RAAA repairs but OSR remained the dominant repair approach for RAAA.

Few studies have investigated the overall and approach-specific trends in EAAA by age. In their analysis of Nationwide Inpatient Sample (NIS) data from 2001 to 2006, Schwarze et al^13^ demonstrated stable rates of EAAA repair with a progressive increase in the proportion of repairs conducted by EVAR. These trends remained consistent when grouped by patient age. The authors demonstrated significant decreases in the rate of elective OSR in each age quartile and concomitant significant increases in elective EVAR rate, with the greatest change occurring among patients 85 years and older (113% change; *P* < .001).^13^ Park et al^14^ demonstrated continuation of these age-specific trends using NIS data from 2005 to 2009.

The findings in this study were generally consistent with the literature on this topic. In this analysis, patients older than 79 years experienced a large increase in EAAA repair that was associated with a greater than 15-fold increase in the uptake of EVAR. These findings may demonstrate an increase in the treatment of comorbid patients that would otherwise be refused AAA repair with OSR. In this scenario, concern about long-term graft durability, reintervention, and secondary rupture are offset by a relatively short life expectancy. However, the multicenter EVAR-2^15^ randomized clinical trial comparing EVAR with no intervention in 338 patients unfit for ruptured OSR demonstrated no difference in all-cause or aneurysm-related mortality for up to 4 years after randomization. The authors also showed higher complication and reintervention rates among patients receiving EVAR and considerably higher costs associated with EVAR. However, the participants of the EVAR-2 study demonstrated low aspirin and statin use, and these findings may reflect EVAR uptake in a population of patients with better medical management and thus lower perioperative risk.^16,17^

The literature regarding the sex-specific trends in EAAA repair is similarly sparse. However, assuming proportionality between that EAAA repair rates and AAA prevalence, the findings in this study with respect to the overall EAAA repair rates grouped by sex are consistent with AAA screening studies, which demonstrate a 4- to 6-fold higher prevalence of AAA among men than women.^1,2,3,4^ Regarding the uptake of elective OSR and elective EVAR by sex, these findings contradict the few data available. Dillavou et al^18^ demonstrated that the uptake of elective EVAR among women lagged behind men in their analysis of Medicare data from 2000 to 2003 (28% in women vs 44.3% in men of all EAAA repairs; *P* < .001). In contrast, this study demonstrated approximately equal (approximately 5%) proportions of elective EVARs among men and women in 2003 and significant increases in elective EVAR uptake in both sexes, with a greater percent change among women. The contrast in the absolute proportions of elective EVAR by sex may be the result of only 1 year of data overlap and a lag in the adoption of EVAR in Canada. Furthermore, despite the almost 16-fold increase in elective EVAR rate among women, EVAR composed about 50% of all EAAA repairs in women in 2016, whereas it has been the predominant approach to EAAA repair in men since approximately 2010. Reasons for this lag in uptake among women may include continued access artery size and tortuosity limitations among women.^19^ Furthermore, despite smaller grafts, studies have demonstrated that women are less likely to meet manufacturer instructions for endograft use owing to shorter proximal neck lengths and greater angulation between neck and aneurysm axis.^20^ In light of these considerations, there may be apprehension about the benefits of EVAR in women owing to the underrepresentation of women in major randomized clinical trials comparing elective EVAR with elective OSR,^7,21,22^ especially considering that population-based studies have demonstrated higher anatomically driven 30-day mortality among female EVAR patients.^23^

The findings of this study regarding overall rates of RAAA repair are consistent with existing Canadian, US, and European studies demonstrating slight declines in RAAA repair rates.^14,24,25,26^ However, given the recognized importance of AAA screening by societal guidelines,^27^ greater differences were expected in the changes in rates among the eldest patients. Guidelines recommend the screening of patients from age 65 to 75 years.^27,28,29^ If screening programs were successful, lower RAAA rates in age quintiles 2 through 5 would have been expected. Although there were greater reductions in RAAA repair rates in age quintiles 2 through 4 and a slower rate reduction in quintile 1 (such that the rate of RAAA repair in this quintile exceeded that in quintiles 2 through 4 in 2016), the rate of RAAA repair in quintile 5 was the highest in both 2003 and 2016. An effective screening program should have had the greatest effect on RAAA reduction in the eldest group of patients owing to a 10-year period during which AAA could have been discovered and treated. However, despite the existence of multiple guidelines recommending AAA screening, Ontario does not have a structured AAA screening program in place, and the uptake of screening according to these guidelines is unknown. Furthermore, guidelines recommend screening in men only. Therefore, the relatively modest decline in RAAA repair in patients older than 79 years may potentially be explained by poor screening uptake and perhaps by unaddressed rupture rates among women. Finally, this modest decline may be the result of the growing availability and experience with ruptured EVAR, resulting in a reduction in the turndown rate for RAAA repair in the eldest patients and those with the highest operative risk. The uptake of ruptured EVAR was the greatest in quintile 5 in this study. However, the effect of ruptured EVAR on RAAA repair turndown is unknown in this study population.

In contrast to data regarding RAAA trends by age group, more data are available regarding these trends by sex. The study by Dillavou et al^18^ demonstrated a greater reduction in overall RAAA repairs among men than women from 1994 to 2003 (29.3% decrease in men vs 12.2% decrease in women). These trends were reiterated in the study by Mureebe et al,^30^ which showed a 52% reduction in men and a 36% reduction in women for RAAA repair. In contrast, this study demonstrated a greater reduction in RAAA repair rates among women (79%) than men (67%). Furthermore, this study demonstrated that a greater proportion of RAAA repairs in women were by EVAR than by OSR in 2016. The greater application of EVAR for RAAA among women than men is in line with results of the Immediate Management of the Patient with Ruptured Aneurysm: Open vs Endovascular Repair (IMPROVE) randomized clinical trial,^31^ which investigated ruptured OSR vs ruptured EVAR in 613 patients. This trial demonstrated no difference in 30-day or 1-year mortality between treatment approaches (30-day mortality: odds ratio [OR], 0.94; 95% CI, 0.67-1.33) but demonstrated a significant interaction effect with sex (*P* = .02) and a significant reduction in mortality risk among women at 30 days (OR, 0.44; 95% CI, 0.22-0.91) that persisted to 1 year (OR, 0.42; 95% CI, 0.21-0.89). Owing to these findings, it is likely that ruptured EVAR will continue to play a greater role in the management of RAAA for women.

### Limitations

This study had several limitations. Administrative and billing codes were used to indirectly identify the cohorts. Although the code combinations were previously validated, the reabstraction methodology only allowed for the measurement of the positive predictive value of the codes. As a result, this study may underrepresent the number of AAA repairs conducted during the study period. Next, this study may have limited generalizability because Ontario administrative data were used. Ontario residents are part of a single-payer, publicly funded health care system in which judicious use of limited health care resources is necessary for distributive justice of these resources. Also, the Ontario age- and sex-specific populations were used to calculate the rates of AAA repair within each subgroup as opposed to using the prevalent AAA population as the denominator for our calculations. This methodology assumes a stable AAA incidence to make inferences regarding AAA repair rate changes and is in line with findings from contemporary incidence studies.^32^ Regardless, further collaborative work must be conducted to investigate the incidence and prevalence of AAA in multiple large North American data sets, as well as to characterize the rates of AAA repair turndown and how these rates have changed in the context of evolving endografts and experience.

## Conclusions

Our population-level time-series analysis of more than 20 000 AAA repairs revealed an overall shift toward EVAR in all age and sex subgroups undergoing EAAA and RAAA repair. The greatest increases were observed among patients undergoing EAAA repair who were older than 79 years and among women. However, the proportion of EAAA repairs among women that were conducted by elective EVAR was still lower than that among in men. These findings suggest a potential need to more judiciously apply elective EVAR in the eldest and most comorbid patients, considering evidence from trials. Our results may also suggest use of continued graft-related restrictions for more widespread application of EVAR to AAA in women. This study may establish a practice pattern for future studies investigating the outcomes of EVAR vs OSR in this large comprehensive data set as endografts and experience continue to evolve.
